# Diagnosing depression in primary care: a Rasch analysis of the major depression inventory

**DOI:** 10.1080/02813432.2019.1568703

**Published:** 2019-01-28

**Authors:** Kaj Sparle Christensen, Eva Oernboel, Marie Germund Nielsen, Per Bech

**Affiliations:** a Research Unit for General Practice and Section for General Medical Practice, Department of Public Health, Aarhus University, Aarhus, Denmark;; b Research Clinic for Functional Disorders and Psychosomatics, Aarhus University Hospital, Aarhus, Denmark;; c Psychiatric Research Unit, Mental Health Centre North Zealand, University of Copenhagen, Copenhagen, Denmark

**Keywords:** Depression, Diagnosis, Mass Screening, Primary Health Care, Psychiatric Status Rating Scales, Psychometrics

## Abstract

**Objective:** This study aims to assess the measurement properties of the Major Depression Inventory (MDI) in a clinical sample of primary care patients.

**Design:** General practitioners (GPs) handed out the MDI to patients aged 18–65 years on clinical suspicion of depression.

**Setting:** Thirty-seven general practices in the Central Denmark Region participated in the study.

**Patients:** Data for 363 patients (65% females, mean age: 49.8 years, SD: 17.7) consulting their GP were included in the analysis.

**Main outcome measures:** The overall fit to the Rasch model, individual item and person fit, and adequacy of response categories were tested. Statistical tests for local dependency, unidimensionality, differential item functioning, and correct targeting of the scale were performed. The person separation reliability index was calculated. All analyses were performed using RUMM2030 software.

**Results:** Items 9 and 10 demonstrated misfit to the Rasch model, and all items demonstrated disordered response categories. After modifying the original six-point to a five-point scoring system, ordered response categories were achieved for all 10 items. The MDI items seemed well targeted to the population approached. Model fit was also achieved for core symptoms of depression (items 1–3) and after dichotomization of items according to diagnostic procedure.

**Conclusion:** Despite some minor problems with its measurement structure, the MDI seems to be a valid instrument for identification of depression among adults in primary care. The results support screening for depression based on core symptoms and dichotomization of items according to diagnostic procedure.

**Key points**
- The Major Depression Inventory (MDI) is widely used for screening, diagnosis and monitoring of depression in general practice.
- This study demonstrates misfit of items 9 and 10 to the Rasch model and a need to modify the scoring system
- The findings support screening for depression based on core symptoms and dichotomization of items according to diagnostic procedure.
- Minor problems with measurement structure should be addressed in future revisions of the MDI.

## Introduction

The 10-item depression scheme Major Depression Inventory (MDI) is widely used in general practice in Denmark [1]. The MDI was originally developed in Danish [2], but it has been translated into several languages, including English.

The MDI is intended to be used both as a *diagnostic* instrument using the algorithms leading to the ICD-10 and DSM-IV categorization of depression [3] and as a *measuring* instrument in which the total score is considered a sufficient statistic for monitoring the level of depression [4].

The MDI is a self-report checklist, which includes: (1) feeling sad, (2) loss of interest, (3) lack of energy, (4) lack of self-confidence, (5) feelings of guilt, (6) feeling that life is not worth living, (7) concentration problems, (8) feeling restless/slowed down, (9) sleeping problems, and (10) reduced/increased appetite. Patients are asked to what extent the symptoms have been present during the last two weeks. Items are completed on a 6-point Likert scale with the response options: (0) “at no time”, (1) “some of the time”, (2) “slightly less than half of the time”, (3) “slightly more than half of the time”, (4) “most of the time”, and (5) “all the time”. Items 8 and 10 are divided into two sub-items, and only the highest score on each sub-item is used.

To study depression severity in relation to treatment outcome, a simple sum of the ten items is used (range: 0–50). The three core items of the MDI (items 1–3) are considered a sufficient measure of screening for depression [1]. In order to diagnose depression according to the ICD-10 criteria, items 1–3 are dichotomized between response categories 4 and 5, whereas items 4–10 are dichotomized between response categories 3 and 4.

The Rasch model is considered a valuable reference standard for several reasons: it has no assumption of normal distribution of data, it can include data on an ordinal scale, and the model provides formal representation of a perfect scale. If data fits the Rasch model, the scale possesses criterion-related construct validity, unidimensionality, additivity, specific objectivity, sufficiency and reliability [5,6]. A consequence of the principle of specific objectivity is that the estimated difference in ability between two people is independent of the difficulty of any particular test item used to compare them [7]. In the Rasch model, the response to any particular item is a function of the difference between the estimated ability of the person (e.g. the level of depression) and a specific characteristic of the item; this represents the difficulty of the item (e.g. the level of depression implied by the item) on a continuous latent variable. The Rasch model assumes that the “easier” the item is to endorse, the more likely it will be affirmed. Likewise, the more affected the respondent is, the more likely the respondent will be to affirm an item compared to a less affected individual.

The MDI has not previously been evaluated using Rasch analysis on a clinical sample of adults in primary care. We set out to address this. More specifically, the analysis aimed to assess the model fit of three subscale scores to the Rasch model: the MDI-10 (summary score used for monitoring), the MDI-3 (core symptoms used for screening), and the dichotomized version of the MDI-10 (used for diagnostics).

## Material

The data material was based on a primary care study aiming to assess the effectiveness of depression screening using the MDI [8]. A total of 440 GP practices in the Central Denmark Region were invited to participate in the study. Of these, 77 (17.5%) volunteered to participate. Due to financial restrictions, a random sample of 50 practices from the volunteers was included. The GPs in these practices participated in the study from 1 October to 1 December 2008. Patients who were able to read and write Danish were eligible for inclusion. All GPs were free to do either 1) case-finding (testing for depression on clinical indication) or 2) screening (routine screening or screening of specific risk groups, e.g. persons with diabetes or heart disease). This study was based on the case-finding sample in order to reflect daily practice and to ensure the clinical validity of our findings. Data were not sampled according to random selection as this is not necessary for conducting the Rasch analysis.

## Data analysis

Descriptive statistics were used to display the clinical characteristics and the demographics of the study population. The MDI data were analyzed using RUMM2030 software [9] to test whether the pattern of item responses observed in the data matched the assumptions of the Rasch measurement model [7]. Due to the consistent polytomous structure (i.e. more than two response categories) of the MDI, the initial step in the Rasch analysis was to conduct a likelihood ratio test. This determined which mathematical derivation of the Rasch model was more appropriate for the data set. The restricted rating scale model (Masters 1982) assumes the distance between item thresholds to be equal across items, whereas the unrestricted partial credit model (Andrich 1988) [10] allows for different distance of thresholds between items. A significant result for the likelihood ratio test (*p* < .05) rejects the use of the restricted rating scale model and supports the use of the unrestricted partial credit model instead.

The following fundamental aspects of Rasch analysis were assessed:

1. *Overall fit to the model*: The overall fit was evaluated using the total chi-square item-trait interaction statistics for the MDI [11,12]. A non-significant chi-square probability value indicates a good level of overall fit. The item person interaction statistics summarizes the individual item fit and the person fit to the model. These standardized fit residual values approximate a z-score. Therefore, a perfect fit would result in a mean value of 0 and a standard deviation of 1 [11]. These summary residual statistics (and deviations from the perfect values) may give an overall impression of the fit, but they do not reveal specific item-level or person-level misfit.
2. *Adequacy of the response categories*: Threshold maps and category probability curves were examined to identify disordered thresholds as a potential cause of misfit [12]. A threshold is the point between two adjacent response categories at which the probability of the respondent endorsing either of the two options is 50% (e.g. equally likely to score “1” or “2”) [13]. A disordered threshold indicates that a response category is never the most likely response at any underlying level of the trait in question. This implies that the original response categories are not functioning as intended, which may be due to a number of reasons [11]. For example, assessors may find it difficult to differentiate between the various response categories for this particular item. The Rasch model is the only IRT model that allows testing of response categories, whereas other IRT models assume correct ordering [12]. When disordered response categories were encountered, categories were collapsed for all items, rescored to adjust for the apparent disorder, and retested to examine how this affects the fit to the Rasch model.
3. *Individual item and person fit*: Standardized fit residual values for items and persons were examined for any indication of misfit (values outside ± 2.5). The residual value is the deviation from the Rasch model summated for each individual item or person [11]. Standardized fit residuals are calculated as differences between observed and calculated responses divided by the standard deviation of the calculated responses. Individual item chi-square fit statistics were also assessed using a Bonferroni-adjusted alpha level [12].
4. *Local dependency*: The response to any one item is dependent on the response to any other item after controlling for the underlying trait. To investigate the local dependency between items, a residual correlation value of more than 0.2 above the average of all item residual correlations was considered indicative of local dependence [14].
5. *Unidimensionality*: To determine whether the scale was measuring a single unidimensional construct, a principal component analysis of the residuals was conducted to identify the two most different subsets of items (i.e. the most positively and negatively factor loading items on the first component). Paired t-tests were performed to compare the scores on the two subsets of items for each person in the sample [15]. If more than 5% of the t-tests were significant (i.e. if the lower 95% confidence limit exceeded 5%), the scale was not considered to be unidimensional.
6. *Differential item functioning (DIF)*: DIF is a certain form of item bias that can occur when different patient groups within the sample (e.g. males and females), despite equal levels of the underlying trait, respond differently to an item. DIF was examined for each item with respect to age (dichotomized at a median of 55 years) and gender using analysis of variance with a Bonferroni-adjusted alpha level [11]. When one subgroup (e.g. females) consistently scores differently on an item across all levels of the trait, this is known as uniform DIF. When the DIF varies across levels of the trait, this is known as non-uniform DIF.
7. *Targeting of the scale*: Targeting of the instrument is assessed by comparing the mean location score for persons with the mean value of zero set for the difficulty of the items. For a well targeted scale, the mean location for persons would be close to zero, which is indicated by inspection of the person-item threshold distribution map [11,12].
8. *Person separation reliability index (PSI)*: The PSI is examined to assess the internal consistency reliability of the scale and the ability of the measure to discriminate amongst persons with different levels of the underlying trait. Interpretation is comparable to Cronbach’s alpha coefficient, where the minimum values of 0.7 and 0.85 indicate acceptable reliability for group and individual use, respectively [12].

## Results

### Sample

In this study, we included persons aged 18–60 years. Of the 363 respondents, 65.0% were female. Mean age was 49.8 years (standard deviation (SD) = 17.7); mean for men = 50.9, mean for women = 49.5, *p* = .49. According to the ICD-10 diagnostic algorithm, 57% (*n* = 207) of the sample were diagnosed with depression.

### Type of rasch model

Of the 363 respondents, three were excluded due to extreme scores and 10 due to missing values. Extreme scores were excluded from the analysis as they provide no information on rank ordering of persons and items. Rasch analysis can handle missing data, but as non-response may indicate lack of engagement with questions, we chose to exclude missing data from our analysis. This left us with 350 records available for analysis. The likelihood ratio test, investigating the hypothesis that the partial credit model fits no better than the rating scale model, was significant (*p* < .00001) for the MDI, thus supporting the use of the unrestricted partial credit model in this study.

### Fit to the rasch model

Initial analysis of the MDI revealed a significant item-trait interaction statistic (*χ*^2^ = 117.32, degrees of freedom (d.f.) = 90, *p* = .028), indicating misfit to the model (Table 1, Analysis 1). Summary fit residual SDs for items (SD = 2.06) and persons (SD = 1.23) were within acceptable limits. The initial analysis fit statistics for each individual item are presented in Table 2. This indicates that item 9 is problematic in terms of the *χ*^2^ statistic and item fit residual and that item 10 is problematic in terms of item fit residual.

**Table 1.** Model fit statistics for MDI items.

Action	Analysis	Overall model fit	Items fit residual Mean (SD)	Persons fit residual Mean (SD)	PSI	Significant t-tests (%) (CI 95%)
Case-finding, *N* = 350	1	*χ*^2^(90) = 117.32, *p* = .028	0.30 (2.06)	−0.30 (1.23)	0.88	7.65 (5.15–11.03)
Rescoring all items, *N* = 350	2	*Χ*^2^(90) = 126.40, *p* = .007	0.32 (2.08)	−0.34 (1.33)	0.88	7.65 (5.15–11.03)
Excluding item 9, *N* = 350	3	*Χ*^2^(90) = 83.94, *p* = .389	0.26 (1.50)	−0.33 (1.19)	0.88	4.53 (2.64–7.32)
Core symptoms: items 1–3, *N* = 338	4	*χ*^2^(24) = 29.92, *p* = .187	−0.29 (0.76)	−0.43 (0.96)	0.76	6.23 (3.88–9.34)
Diagnostic dichotomization, *N* = 276	5	*χ*^2^(80) = 87.25, *p* = .271	−0.03 (1.43)	−0.06 (0.69)	0.72	2.83 (1.26–5.63)

*χ*^2^(df): chi-square (degrees of freedom); p: probability; SD: standard deviation; PSI: person separation index (with extremes); CI: Confidence Interval. All analyses are separate analyses. Subsequent reduction in N is due to exclusion of extreme scores from the analysis, as they provide no information on rank ordering of persons or items.

**Table 2.** Individual item fit for MDI items.

	Response category,^a^*n*(%)						Logit location	s.e.	Fit residual	*χ*^2^	*d.f.*	*χ*^2^ probability
MDI item	0	1	2	3	4	5							
1 Have you felt low in spirits or sad?	12(3)	75(21)	34(10)	71(20)	120(34)	38(11)	−0.347	0.052	−1.550	11.537	9	0.241
2 Have you lost interest in your daily activities?	37(11)	61(17)	39(11)	54(15)	117(33)	42(12)	−0.080	0.048	−1.313	12.293	9	0.197
3 Have you felt lacking in energy and strength?	13(4)	41(12)	33(9)	43(12)	127(36)	93(27)	−0.721	0.052	−0.295	6.855	9	0.652
4 Have you felt less self-confident?	46(13)	60(17)	42(12)	58(17)	82(23)	62(18)	−0.064	0.045	0.376	8.072	9	0.527
5 Have you had a bad conscience or feelings of guilt?	59(17)	65(19)	36(10)	57(16)	80(23)	53(15)	0.094	0.044	0.069	6.019	9	0.738
6 Have you felt that life wasn’t worth living?	140(40)	85(24)	34(10)	41(12)	30(9)	20(6)	0.842	0.046	−0.295	5.244	9	0.813
7 Have you had difficulty in concentrating, e.g. when reading the newspaper or watching television?	65(19)	81(23)	42(12)	65(19)	68(19)	29(8)	0.346	0.046	0.665	7.720	9	0.563
8 Have you felt very restless?/ Have you felt subdued or slowed down?	20(6)	61(17)	36(10)	68(19)	110(31)	55(16)	−0.339	0.051	−2.064	16.659	9	0.054
9 Have you had trouble sleeping at night?	48(14)	74(21)	39(11)	41(12)	74(21)	74(21)	−0.053	0.043	4.810	35.106	9	0.000
10 Have you suffered from reduced appetite?/ Have you suffered from increased appetite?	81(23)	78(22)	41(12)	48(14)	61(17)	61(12)	0.323	0.043	2.576	7.815	9	0.553

a Response categories: (0) at no time; (1) some of the time; (2) slightly less than half of the time; (3) slightly more than half the time; (4) most of the time; (5) all the time.

### Adequacy of the response categories

**Figure 1.**
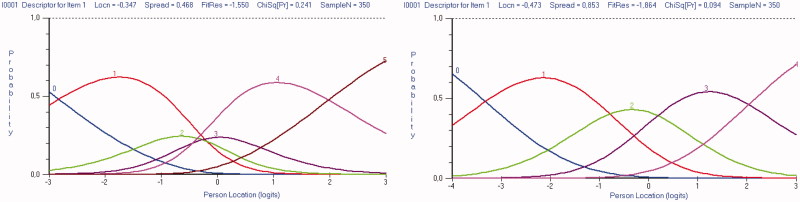
The MDI category probability curves for item 1 ‘feeling sad’ displaying disordered six-point response categories and corrected five-point response categories.

**Figure 2.**
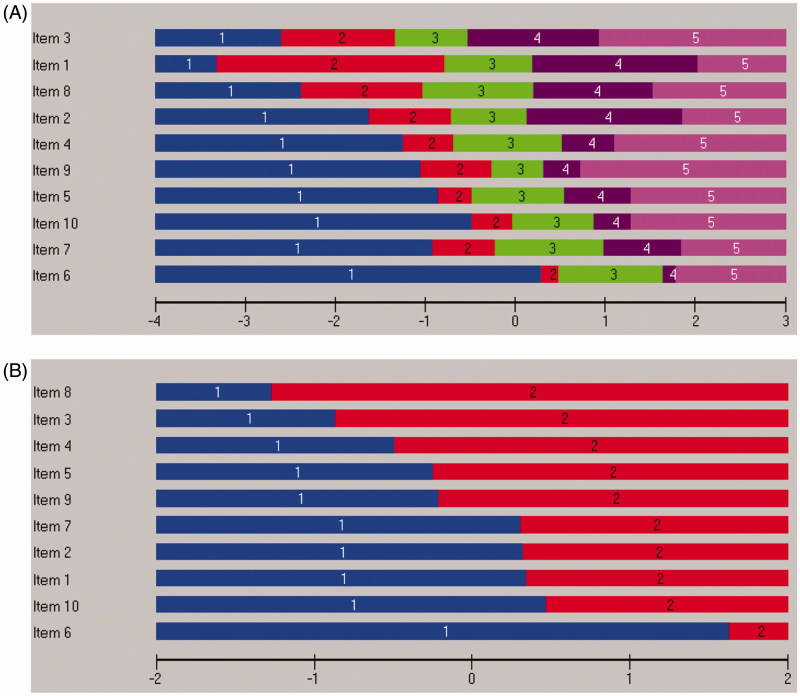
All 10 MDI items displayed disordered six-point response categories and corrected five-point response categories (A). Dichotomization according to diagnostic algorithm revealed ordered response categories (B). All items are sorted in location order.

Inspection of the category probability curves demonstrated disordered response thresholds for all 10 items. The curves indicated that assessors could not truly differentiate between response categories 2 “slightly less than half of the time” and 3 “slightly more than half of the time” on the original six-point scale (Figure 1). However, creating a five-point scoring system for all items by collapsing these two response categories into a single category resulted in ordered thresholds for all ten items, but without significantly improving the fit to the model (Table 1, Analysis 2). This is graphically illustrated in Figure 2(A), which shows that each response category, as the level of trait increases, has a point along the level of trait at which it is the most likely response category to be endorsed.

### Local independency

Indication of local dependency between items was found as the residual correlations were above 0.2 for item pairs, including items 9 and 10 (Supplementary data, Table S1).

### Unidimensionality

Testing for dimensionality revealed significant t-tests outside the critical value of 5%, which indicates that the MDI may not be a unidimensional construct (Table 1, Analysis 1–2). When we excluded item 9, both the item with the largest item fit residual and the overall item fit improved, and t-test values fell within the 5% limit; this suggests unidimensionality of the MDI (Table 1, Analysis 3).

### Scale reduction and dichotomization of items

Model fit was achieved when the scale was reduced to core symptoms (items 1–3) of depression (Table 1, Analysis 4). Dichotomization of response categories according to diagnostic criteria (000011 for items 1–3 and 000111 for items 4–10) demonstrated no significant effect on the overall item fit statistics (Table 1, Analysis 5). Diagnostic dichotomization of items resulted in ordered response categories as illustrated in Figure 2(B).

### Differential item functioning

The MDI demonstrated no item bias (DIF) with respect to sex and age (Supplementary data, Tables S2 and S3).

### Targeting and reliability

**Figure 3.**
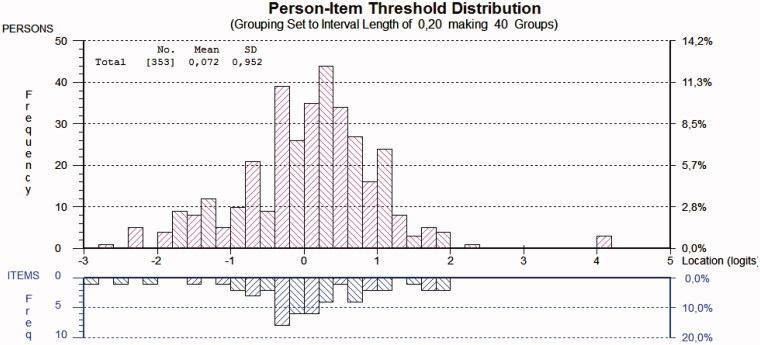
The person-item threshold map for the MDI items for the primary care sample. A total of 57% of persons were classified as clinically depressed according to the MDI algorithm.

Inspection of the person-item distribution map (Figure 3) revealed that the scale was reasonably well targeted (mean person location was 0.072; SD = 0.952). The easiest item to endorse was item 3 “lack of energy”, and the hardest item to endorse was item 6 “feeling that life is not worth living”. Person separation reliability was well above the acceptable limit (0.80), indicating that the MDI could reliably distinguish between different persons.

## Discussion

To our knowledge, this is the first study to perform Rasch analysis on the MDI in a clinical population of primary care patients. The findings of this study have shown that item 9 (sleep problems) and item 10 (appetite problems) demonstrated misfit to the Rasch model. Our analyses revealed that all ten items demonstrated disordered response categories. After modifying the original six-point scoring system to a five-point system, ordered response categories were achieved for all ten items. Dichotomization of items according to the ICD-10 diagnostic procedure demonstrated ordered response categories for all ten items. The total MDI scale was reasonably well targeted, as demonstrated by an overlap between person ability and item difficulty on the person-item threshold distribution map. The threshold map showed minor clustering of persons at the lower levels of the trait (i.e. floor effect), and only few gaps were found in the spread of both items and persons over the range of the construct.

### Comparison with other studies

Olsen et al. [16] have previously assessed the validity of the MDI in a mixed group of inpatients and outpatients in rheumatic and psychiatric care. Findings from their study suggested the MDI to be rank-ordered and have a unidimensional construct. Conflicting with these results, a recent study by Amris [17] in female patients with chronic widespread pain identified problems with both scalability and unidimensionality of the MDI. In accordance with our findings, Amris and colleagues suggest that response categories 2 and 3 should be collapsed into a new category (termed ‘half of the time’) to achieve sufficient rating scale properties. While Amris et al. suggest exclusion of items 9 and 10, we suggest this issue to be further investigated first. The misfit of items 9 and 10 may be caused by an overlap with symptoms of somatic illness, anxiety, or medication of respondents (DIF). It is likely that comorbidity and medical treatment may affect both sleep and appetite. Therefore, we recommend clinicians to carefully evaluate the impact of comorbid conditions and medical treatment when considering responses to items 9 and 10.

### Limitations

The sample of participants is restricted to a case-finding sample of 18- to 65-year-olds and may not fully represent the diverse characteristics found within a population of patients tested for depression in primary care.

In its current format, the MDI displays minor problems with regard to its measurement structure among a primary care sample of persons tested on clinical indication of depression. However, these problems can be addressed to help ensure development of a more reliable and stable tool. The results also offer support for the MDI-3 as a screening tool for depression and for the dichotomization of items according to the diagnostic algorithm.

### Meaning and implications for clinicians

Our findings support screening for depression by simply asking about three core symptoms: 1) Do you feel sad? 2) Have you lost interest in things? and 3) Do you feel lack of energy? If the responses to at least two of these questions are positive (“Yes”), the patient should be encouraged to fill in the entire MDI questionnaire. Dichotomization of responses according to scoring procedure provides a good basis for ranking of depression severity. Patients scoring with depression should be further clinically assessed and provided with further resources. A total summary score may be useful for monitoring purposes, but minor adjustment of response categories and modification or exclusion of items 9 and 10 should be considered in future revisions of the MDI.

## Supplementary Material

Supplemental Material
